# Long-term outcomes and factors associated with visual recovery after small-gauge vitrectomy for infectious endophthalmitis: a 10-year experience at a single tertiary center in Taiwan

**DOI:** 10.1007/s00417-026-07279-0

**Published:** 2026-05-14

**Authors:** Chung-Hao Hsiao, Hung-Ju Chen, Chia-Jen Chang, Shih-Chao Feng, Yuan-Shao Cheng, Wen-Jung Lo

**Affiliations:** 1 Department of Ophthalmology, Taichung Municipal Geriatric Rehabilitation General Hospital, Taichung, Taiwan; 2 Department of Ophthalmology, Taichung Veterans General Hospital, Taichung, Taiwan; 3 Department of Ophthalmology, School of Medicine, National Yang Ming Chiao Tung University, Taipei, Taiwan; 4 Program for Precision Health and Intelligent Medicine, Graduate School of Advanced Technology, National Taiwan University, Taipei, Taiwan

**Keywords:** Endophthalmitis, Pars plana vitrectomy, Microincision vitrectomy surgery (MIVS), Visual prognosis

## Abstract

**Purpose:**

To evaluate long-term visual outcomes and prognostic factors after small-gauge pars plana vitrectomy (PPV) for exogenous endophthalmitis.

**Methods:**

Single-center, retrospective cohort of 45 eyes (45 patients) undergoing PPV for exogenous endophthalmitis (June 2014–September 2024). Demographics, etiology, microbiology, surgical parameters, and outcomes were reviewed. Best-corrected visual acuity (BCVA) was recorded preoperatively and at final follow-up. Associations with final BCVA were explored using univariate tests.

**Results:**

Median BCVA improved from hand motion (~ 20/4000; logMAR 2.30) to approximately 20/1200 (logMAR 1.78; *p* = 0.004). Overall, 51.1% improved and 24.4% achieved ≥ 20/40. Factors associated with worse final BCVA included poorer initial BCVA (*p* = 0.002), culture positivity (*p* = 0.037), and intraoperative retinal detachment (*p* = 0.009). Favorable factors were 27-gauge PPV (*p* = 0.044) and post-cataract etiology (*p* = 0.006).

**Conclusion:**

Small-gauge PPV, including 27-gauge, was associated with meaningful visual improvement in exogenous endophthalmitis. Final visual outcomes appeared to be influenced by baseline vision, microbial virulence, and the presence of retinal detachment. However, given the highly heterogeneous nature of this cohort, these findings should be interpreted with caution and may warrant further validation. Our findings provide contemporary data complementing the Endophthalmitis Vitrectomy Study and reflect outcomes achievable with modern techniques.

**Supplementary Information:**

The online version contains supplementary material available at https://doi.org/10.1007/s00417-026-07279-0.

## Key messages

*****What is known***:**

- Exogenous endophthalmitis is a severe, vision-threatening intraocular infection most commonly occurring after ocular surgery, intravitreal injection, or penetrating trauma.
- The Endophthalmitis Vitrectomy Study established vitrectomy as a beneficial intervention for severe endophthalmitis; however, the results were derived from an earlier surgical era and may not fully reflect outcomes achievable with contemporary surgical techniques.

*****What is new***:**

- In a real-world 10-year cohort encompassing diverse etiologies beyond post-cataract cases, pars plana vitrectomy resulted in meaningful visual improvement in a substantial proportion of eyes with exogenous endophthalmitis.
- Poor initial visual acuity, male sex, positive vitreous culture, and the presence of retinal detachment were associated with worse final visual outcomes, whereas post-cataract etiology was associated with a more favorable prognosis.
- Modern small-gauge vitrectomy appears to be a viable surgical option for exogenous endophthalmitis, although severe visual deterioration may still occur in high-risk cases.

**Supplementary Information:**

The online version contains supplementary material available at https://doi.org/10.1007/s00417-026-07279-0.

## Introduction

Exogenous endophthalmitis is a severe and potentially blinding intraocular infection that most commonly occurs following ocular surgery, intravitreal injection, or penetrating trauma [1]. Despite advances in antimicrobial therapies and surgical techniques, prognosis remains guarded, particularly in cases of delayed presentation or infection by virulent pathogens [2]. Early and effective management is crucial to preserve vision and prevent irreversible structural damage to ocular tissues.

Pars plana vitrectomy (PPV) has evolved as a pivotal intervention for endophthalmitis, enabling removal of infectious material, inflammatory debris, and vitreous opacities, while facilitating intraocular antibiotic delivery. Since the landmark Endophthalmitis Vitrectomy Study (EVS) [3, 4], which demonstrated the benefits of vitrectomy in selected post-cataract cases, the role of PPV has expanded to encompass a broader range of clinical scenarios. However, the EVS included only patients with visual acuity (VA) of light perception or better, and excluded those with no light perception (NLP) or with etiologies such as post-vitrectomy, post-injection, or bleb-associated infections, thereby limiting its applicability in today’s diverse clinical landscape [5].

Recent advances in microincision vitrectomy (MIVS), including 27-gauge systems, have improved surgical control through enhanced fluidics [6], reduced surgical invasiveness due to smaller incision size [7], and have been associated with favorable visual outcomes in selected vitreoretinal surgeries [8, 9]. Nonetheless, evidence regarding visual outcomes and prognostic factors following PPV for exogenous endophthalmitis, especially in the era of MIVS, remains limited and inconclusive [3].

In this study, we retrospectively analyzed a 10-year cohort of patients who underwent PPV for exogenous endophthalmitis at a tertiary referral center in Taiwan. We aimed to assess surgical outcomes, identify associated factors influencing final best-corrected visual acuity (BCVA), and evaluate the impact of surgical variables such as gauge selection and timing.

## Materials and methods

### Study design, population, data collection and definitions

This is a retrospective, single-center study that was approved by the Institutional Review Board of the Taichung Veterans General Hospital (CE25531A). The need for informed consent from study participants was waived by Institutional Review Board of the Taichung Veterans General Hospital and the study procedures adhered to the tenets of the Declaration of Helsinki. Patients who underwent PPV for exogenous endophthalmitis at Taichung Veterans General Hospital were consecutively enrolled from June 2014–September 2024.

Medical records were reviewed, and data were collected on the following parameters: age, gender, laterality, history of intraocular surgeries, prior ocular diseases, history of ocular trauma, etiology of endophthalmitis, cultured pathogens, presenting symptoms, time interval from presentation to PPV, number of intravitreal antibiotic injections, and preoperative ocular assessments including BCVA, intraocular pressure (IOP), slit-lamp biomicroscopy, and fundus examination. Additional data included intraoperative findings, postoperative complications, subsequent surgical interventions after the initial PPV, BCVA at the final visit, and follow-up duration. Patients with a follow-up period of less than three months were excluded from the analysis.

Intravitreal antibiotic injections were administered using 1 mg of vancomycin and 2.25 mg of ceftazidime injected into the vitreous cavity. Urgent intravitreal antibiotic injection was defined as administration at the time of diagnosis. Postoperative complications, including hypotony (IOP ≤ 5mmHg) [10], ocular hypertension (IOP ≥ 25 mmHg) [11] and cystoid macular edema (CME), were recorded. CME was identified using optical coherence tomography (OCT). The primary outcome measure was BCVA at the final visit. Secondary outcomes included the identification of associated factors influencing final BCVA. For statistical conversion, the following logMAR values were applied: NLP = 2.9; light perception (LP) = 2.6; hand motion (HM ≈ 20/4000; logMAR 2.30); counting fingers (CF ≈ 20/2000; logMAR 2.0) [12].

### Surgical technique

All patients underwent standard three-port PPV performed by experienced vitreoretinal surgeons. The gauge of the PPV was selected at the surgeon’s discretion. If the surgical view was obscured by hypopyon or exudates in the anterior chamber, anterior chamber irrigation was performed prior to PPV. For post-cataract endophthalmitis, intraocular lens removal was performed based on the surgeon’s judgement.

Following core vitrectomy and induction of posterior vitreous detachment, peripheral vitreous shaving was performed as extensively as possible. Silicone oil was used as a vitreous tamponade in eyes with rhegmatogenous retinal detachment (RD) or at the surgeon’s discretion in cases with severe intraocular inflammation where undetected retinal breaks might be present. At the end of surgery, an intravitreal injection of antibiotics (1 mg vancomycin and 2.25 mg ceftazidime) was administered. Sclerotomies were sutured with 8 − 0 vicryl suture in cases of sclerotomy leakage or when silicone oil tamponade was applied.

If RD was identified during PPV, all retinal breaks were carefully located, and any associated vitreous traction was removed. Air–fluid exchange was performed to evacuate subretinal fluid, followed by endolaser treatment to the retinal breaks. None of the eyes received intravitreal or periocular steroids. Postoperatively, topical antibiotics (ampicillin 50 mg/mL and ceftazidime 50 mg/mL) were administered. Topical corticosteroids were prescribed based on the patient’s clinical condition.

### Statistical analysis

Data were analyzed using IBM SPSS Statistics (International Business Machines Corp, New York, USA). Continuous variables are presented as mean ± standard deviation (SD), and categorical variables as numbers and percentages. Nonparametric tests were used for all analyses. Paired continuous variables were analyzed using the Wilcoxon signed-rank test. Associated factors for visual outcomes were assessed using the Mann–Whitney U test or Kruskal–Wallis test, as appropriate, with results presented as median and interquartile range (IQR). Correlations were tested with Spearman’s rho correlation coefficient. A two-tailed p-value of less than 0.05 was considered statistically significant. All statistical analyses of associated factors for visual outcomes were conducted as exploratory analyses. Given the retrospective design and the limited sample size, multiple univariable comparisons were performed without adjustment for multiplicity. These analyses are intended solely for exploratory purposes and should not be interpreted as establishing predictive or causal relationships.

## Results

### Baseline characteristics

A total of 45 patients with 45 eyes were included in this study. The mean age was 64.2 ± 11.7 years, ranging from 36 to 86 years. Among them, 24 were male (53.3%) and 21 were female (46.7%). The right eye was affected in 25 cases, accounting for 55.6%. Twenty patients (44.4%) had diabetes mellitus. Comorbidities prior to PPV included diabetic retinopathy in 4 eyes (8.9%), glaucoma in 3 eyes (6.7%), previous PPV for epiretinal membrane (ERM) in 1 eye (2.2%), diabetic macular edema treated with intravitreal anti-vascular endothelial growth factor (VEGF) injections in 3 eyes (6.7%), prior PPV for macular hole in 1 eye (2.2%), central retinal vein occlusion treated with intravitreal anti-VEGF injections in 1 eye (2.2%), and a history of open globe injury in 1 eye (2.2%). The overall median follow-up duration was 516 days (IQR 144–1262.5). Detailed demographic data are presented in Table 1.

**Table 1:** Patient Characteristics

Age (years)	
Mean ± SD	64.2 ± 11.7
Gender, no. (%)	
Male	24 (53.3%)
Female	21 (46.7%)
Laterality, no. (%)	
OD	25 (55.6%)
OS	20 (44.4%)
Diabetes mellitus	20 (44.4%)
Comorbidity, no. (%)	
Diabetic retinopathy	4 (8.9%)
Glaucoma	3 (6.7%)
ERM status post PPV	1 (2.2%)
History of DME status post IVI anti-VEGF	3 (6.7%)
Macular hole status post PPV	1 (2.2%)
History of CRVO status post IVI	1 (2.2%)
History of OGI	1 (2.2%)
Follow up time (days), median (IQR)	516 (144-1262.5)

*PPV* pars plana vitrectomy, *ERM* epiretinal membrane, *IVI* intravitreal injection, *CRVO* central retinal vein occlusion, *OGI* open globe injury, *DME* diabetic macular edema, *VEGF* vascular endothelial growth factor

### Etiology of infection and pathogens

The most common cause of exogenous endophthalmitis was cataract surgery, accounting for 34 eyes (75.6%), followed by PPV in 6 eyes (13.3%), intravitreal injection in 2 eyes (4.4%), trauma in 1 eye (2.2%), bleb needling in 1 eye (2.2%), and trabeculectomy in 1 eye (2.2%). Vitreous cultures were performed in 39 eyes, representing 86.7% of the cases, and yielded positive results in 17 eyes (43.6%). The most frequently identified pathogen was *Staphylococcus epidermidis*, found in 7 eyes, followed by *Streptococcus pneumoniae*, coagulase-negative Staphylococci, and *Enterococcus faecalis*, each detected in 2 eyes. Details regarding etiology and pathogens are summarized in Table 2.

**Table 2:** Etiology of infection and pathogens

Cause of endophthalmitis, no. (%)	
Cataract surgery	34 (75.6%)
PPV	6 (13.3%)
Intravitreal injection	2 (4.4%)
Trauma	1 (2.2%)
Bleb needling	1 (2.2%)
Trabeculectomy	1 (2.2%)
Vitreous culture performed, no. (%)	39 (86.7%)
Culture result, no. (%) (*n* = 39)	
Growth	17 (43.6%)
No growth	22 (56.4%)
Culture pathogen, no. (%) (*n* = 17)	
Staphylococcus epidermidis	7 (41.2%)
Streptococcus pneumoniae	2 (11.8%)
Coagulase-negative Staphylococci	2 (11.8%)
Enterococcus faecalis	2 (11.8%)
Viridans streptococci	1 (5.9%)
Unidentified Gram-positive bacillus	1 (5.9%)
MRSA	1 (5.9%)
Pseudomonas aeruginosa	1 (5.9%)

*MRSA* methicillin-resistant Staphylococcus aureus

### Presenting clinical features

Presenting clinical features are summarized in Table 3. Most eyes presented with blurred vision and pain, observed in 28 cases (62.2%). The median interval from the inciting event to presentation was 5 days (IQR 4–8). The median initial BCVA was HM (~ 20/4000; logMAR 2.30, IQR 2.00–2.30), and the median preoperative IOP was 14 mmHg (IQR 11–19). At presentation, 73.2% of eyes had a BCVA of HM or worse. Specifically, 51.2% had HM, 17.1% had LP, and 4.9% had NLP. Among the 45 eyes, 41 were pseudophakic and 4 were phakic. Hypopyon was observed in 30 eyes (66.7%), and urgent intravitreal antibiotic injections were administered in 40 eyes (88.9%). The mean number of intravitreal injections throughout the treatment course was 4.22 ± 2.59, including 2.07 ± 1.78 injections administered before PPV and 1.18 ± 1.84 injections after PPV. The median interval from diagnosis to PPV was 3 days (IQR 1–7). Of all cases, 10 eyes (22.2%) underwent PPV within 24 h, 27 eyes (60.0%) within 3 days, and 34 eyes (75.6%) within 7 days. The most commonly used gauge for PPV was 25-gauge, used in 53.3% of cases, followed by 27-gauge in 31.1% and 23-gauge in 15.6%.

**Table 3:** Clinical features and treatment

Symptoms, no. (%)	
Blurred vision and pain	28 (62.2%)
Blurred vision	14 (31.1%)
Pain	3 (6.7%)
Interval between causing event and date of presentation (days), median (IQR)	5 (4–8)
Initial visual acuity (logMAR), median (IQR)	2.30 (2.00-2.30)
Intraocular pressure (mmHg), median (IQR)	14 (11–19)
Lens status, no. (%)	
Phakic	4 (8.9%)
Pseudophakic	41 (91.1%)
Hypopyon	30 (66.7%)
Urgent IVI antibiotics	40 (88.9%)
Total number of IVI, mean ± SD	4.22 ± 2.59
Number of IVI before PPV, mean ± SD	2.07 ± 1.78
IVI when PPV, no. (%)	44 (97.8%)
Number of IVI after PPV, mean ± SD	1.18 ± 1.84
Time interval between presentation to PPV, no. (%)	
≦ 24 h	10 (22.2%)
≦ 3 days	27 (60.0%)
≦ 7 days	34 (75.6%)
> 7 days	11 (24.4%)
PPV gauge, no. (%)	
23G	7 (15.6%)
25G	24 (53.3%)
27G	14 (31.1%)

*IVI* intravitreal injection, *PPV* pars plana vitrectomy;

### Surgical outcomes and complications

Following PPV, the median BCVA significantly improved from HM (~ 20/4000; logMAR 2.30, IQR 2.00–2.30) to approximately 20/1200 (logMAR 1.78, IQR 0.35–2.90; *p* = 0.004). A total of 23 patients (51.1%) showed improvement in BCVA after the procedure. At the final follow-up, 51.1% of eyes achieved a BCVA better than 20/1200, and 24.4% reached 20/40 or better, compared to only 14.6% and 0%, respectively, before PPV. However, the proportion of eyes with NLP increased from 4.9% preoperatively to 31.7% postoperatively.

Among eyes with a final BCVA of NLP, the median presenting BCVA was logMAR 2.60 (IQR, 2.30–2.60). Non–cataract-related endophthalmitis accounted for 50% of cases. The median interval from presentation to PPV was 5 days (IQR, 0–10), with 64.3% of eyes undergoing surgery after 3 days. Cultures were positive in 76.9% of cases, including *Staphylococci* (3 eyes), *Streptococcus pneumoniae* (2 eyes), *Enterococcus faecalis* (2 eyes), unidentified Gram-positive bacilli (1 eye), *Pseudomonas aeruginosa* (1 eye), and methicillin-resistant Staphylococcus aureus (MRSA) (1 eye). One eye with MRSA ultimately required evisceration due to uncontrolled infection. RD was present in 5 eyes (35.7%). Regarding surgical instrumentation, 23-gauge PPV was performed in 3 eyes (21.4%), 25-gauge in 10 eyes (71.4%), and 27-gauge in 1 eye (7.1%).

Regarding the outcomes of eyes that underwent vitrectomy beyond the EVS criteria, eyes presenting with NLP remained NLP at the final follow-up despite undergoing PPV. In contrast, eyes presenting with HM or better demonstrated visual improvement after surgery. The median BCVA improved from logMAR of 2.30 (IQR, 2.00–2.30) to logMAR of 0.85 (IQR, 0.24–2.30). At the final follow-up, 50.0% of these eyes achieved a BCVA of 20/100 or better, and 31.3% achieved 20/40 or better. Overall, BCVA improved or remained stable in 66.7% of eyes in this subgroup.

For the subgroup of non-cataract surgery-related endophthalmitis, the median preoperative and postoperative BCVA were 2.60 (IQR, 2.53–2.68) and 2.90 (IQR, 1.65–2.90) logMAR, respectively. By the final follow-up, 63.6% had NLP, while 36.4% retained HM or better vision. Detailed visual outcomes before and after PPV are summarized in Table 4.

**Table 4:** Visual outcomes

	Initial visual outcomes	Final visual outcomes	*p*- value
BCVA (logMAR), median (IQR)	2.30 (2.00-2.30)	1.78 (0.35–2.90)	0.004**
NLP	4.9%	31.7%	
LP	17.1%	2.4%	
HM	51.2%	7.3%	
CF	12.2%	7.3%	
1/60 to 0.4	14.6%	26.7%	
≥ 0.5	0%	24.4%	

Wilcoxon Signed Ranks test. *p < 0.05, **p < 0.01

BCVA, best corrected visual acuity; NLP, no light perception; LP, light perception; HM, hand motion; CF, counting fingers

Retinal breaks and RD were identified during PPV in seven eyes (15.6%), all of which received silicone oil tamponade. Silicone oil was also used in other cases based on the surgeon’s judgement, with a total of 15 eyes (33.3%) ultimately receiving tamponade. Among eyes with post-cataract endophthalmitis, 9 out of 34 (26.5%) underwent intraocular lens removal during surgery.

The most common complication following PPV was ERM formation, occurring in nine eyes (20%). Other complications included postoperative ocular hypertension in six eyes (13.3%), CME in six eyes (13.3%), hypotony in two eyes (4.4%), secondary glaucoma in two eyes (4.4%), and corneal decompensation in one eye (2.2%). Postoperative RD occurred in four eyes. Among them, one eye underwent repeat PPV with sulfur hexafluoride tamponade, two eyes received PPV with silicone oil tamponade, and one eye was managed with PPV, scleral buckle, and silicone oil tamponade. All four eyes achieved retinal reattachment by the final visit.

Two eyes developed phthisis bulbi; neither had received silicone oil tamponade and both developed postoperative hypotony. A summary of surgical outcomes and complications is provided in Table 5.

**Table 5:** Surgical outcomes and complications

Final best corrected visual acuity (logMAR), median (IQR)	1.78 (0.35–2.90)
Lensectomy or IOL removal when PPV, no. (%)	
Lensectomy	3 (6.7%)
IOL removal	9 (20%)
Vitreous tamponade after PPV, no. (%)	
Balanced Salt Solution	29 (64.4%)
Silicone oil	15 (33.3%)
Sulfur hexafluoride	1 (2.2%)
Retinal detachment (intraoperative find), no. (%)	7 (15.6%)
Retinal break, no. (%)	7 (15.6%)
Complications	
Epiretinal membrane, no. (%)	9 (20%)
Post-operative ocular hypertension, no. (%)	6 (13.3%)
Cystoid macular edema, no. (%)	6 (13.3%)
Post-operative retinal detachment, no. (%)	4 (8.9%)
Hypotony, no. (%)	2 (4.4%)
Secondary glaucoma, no. (%)	2 (4.4%)
Phthisis bulbi, no. (%)	2 (4.4%)
Neovascular glaucoma, no. (%)	1 (2.2%)
Cornea decompensation, no. (%)	1 (2.2%)
Second surgery, no. (%)	12 (26.7%)
Remove silicone oil	2 (4.4%)
PPV	3 (6.7%)
PPV + SB	2 (4.4%)
TSCPC	1 (2.2%)
Anterior chamber irrigation	1 (2.2%)
Cataract surgery or Secondary IOL insertion	2 (4.4%)
Evisceration	1 (2.2%)
Third surgery, no. (%)	
PPV	2 (4.4%)

*IOL* intraocular lens, *PPV* pars plana vitrectomy, *SB* scleral buckling, *TSCPC* Transsclera l cyclophotocoagulation;

### Associated factors for visual outcomes

Worse final BCVA was significantly associated with poor initial BCVA, showing a moderate positive correlation (Spearman’s rho = 0.47, *p* = 0.002), as well as male gender (*p* = 0.042), positive vitreous culture (*p* = 0.037), and the presence of RD during PPV (*p* = 0.009). On the other hand, favorable associated factors included an initial BCVA of HM (~ 20/4000) or better (*p* < 0.01), a post-cataract etiology (*p* = 0.006), and the use of a 27-gauge instrument for PPV (*p* = 0.044). No significant associations were found between final BCVA and age, diabetes mellitus, hypopyon, timing of PPV, or postoperative RD. Eyes that underwent vitrectomy within 3 days of diagnosis had a median final BCVA of logMAR 1.78 (IQR, 0.30–2.30), compared with logMAR 2.34 (IQR, 0.63–2.90) for those treated after 3 days (*p* = 0.230). These findings should be considered exploratory, aimed at identifying potential factors that may influence visual outcomes. Detailed analysis of associated factors is provided in Table 6.

**Table 6:** Associated factors for final visual outcomes

	Final BCVA, median (IQR)	*p*-value
Gender		0.042*
Male	2.30 (0.78–2.90)	
Female	0.70 (0.22–2.45)	
Diabetes mellitus		0.214
Yes	2.00 (0.78–2.90)	
No	0.70 (0.26–2.75)	
Hypopyon		0.149
Yes	2.00 (0.70–2.90)	
No	0.40 (0.00-2.90)	
Initial BCVA ≥ HM		< 0.001**
Yes	0.85 (0.24–2.30)	
No	2.90 (2.90–2.90)	
Time interval between presentation to PPV ≦ 3 days		0.230
Yes	1.78 (0.30–2.30)	
No	2.34 (0.63–2.90)	
Cause of endophthalmitis		0.006**
Cataract surgery	1.00 (0.22–2.38)	
Non-cataract surgery	2.90 (1.00-2.90)	
Culture result		0.037*
Growth	2.90 (0.70–2.90)	
No growth	1.15 (0.38–2.30)	
PPV gauge		0.044*
23G	2.30 (0.30–2.90)	
25G	2.15 (0.70–2.90)	
27G	0.50 (0.04–2.08)	
RD, intraoperative find		0.009**
Yes	2.90 (2.30–2.90)	
No	1.15 (0.28–2.68)	
RD, post- operative		0.764
Yes	1.89 (0.61–2.23)	
No	1.78 (0.35–2.90)	

Mann-Whitney test. Kruskal-Wallis test. *p < 0.05, **p < 0.01

*BCVA* best corrected visual acuity, *HM* hand motion, *PPV* pars plana vitrectomy, *RD* retinal detachment

## Discussion

This decade-long real-world cohort from Taiwan extends the evidence base beyond the EVS by encompassing diverse etiologies such as post-vitrectomy, post-injection, bleb-related, and trauma-associated endophthalmitis, thereby better reflecting the heterogeneity of modern clinical practice.

We observed a statistically significant improvement in BCVA following PPV, with the median Snellen acuity improving from hand motion (~ 20/4000; logMAR 2.30) to approximately 20/1200 (logMAR 1.78; *p* = 0.004). Overall, 51.1% of eyes gained vision, and 24.4% achieved a final BCVA better than 20/40. Compared to the EVS [3], our outcomes are comparable, though not superior (24.4% vs. 33%). This difference may reflect the broader and more heterogeneous etiologies in our cohort, including cases related to prior vitrectomy, intravitreal injections, trauma, and filtering surgeries [13, 14], which are often associated with more complex clinical conditions and more virulent pathogens.

Nevertheless, the NLP rate increased from 4.9% preoperatively to 31.7% postoperatively. Several factors may explain this deterioration. Eyes that progressed to NLP underwent PPV later than those without NLP (median 5 vs. 2 days), with a higher proportion receiving surgery after 3 days (64.3% vs. 29.0%). This is consistent with previous literature suggesting a potential association between earlier vitrectomy and better visual outcomes in endophthalmitis [15]. In addition, culture positivity was also higher in the NLP group (76.9% vs. 26.9%), consistent with previous reports, including Welch et al., who demonstrated worse visual outcomes among 290 patients, of whom 100 experienced severe vision loss [16]. Previous studies have reported that *Enterococcus faecalis* [17], *MRSA* [18], or *Pseudomonas aeruginosa* [19] are associated with poor visual outcomes, and these pathogens were identified exclusively in eyes that progressed to NLP. Additionally, NLP eyes had a higher prevalence of RD (35.7% vs. 6.5%) and more non–cataract-related etiologies (50% vs. 12.9%). Collectively, these factors, including delayed surgery, more virulent pathogens, higher culture positivity, and non–cataract etiology, likely contributed to the increased rate of postoperative NLP. However, given the observational design, no causal relationship between surgical timing and visual prognosis can be established. Nonetheless, these findings suggest that PPV may not always prevent severe vision loss in cases of exogenous endophthalmitis, particularly when multiple risk factors coexist.

We identified several factors significantly associated with poor visual outcomes, including male gender, poor initial BCVA, positive vitreous culture, and RD identified either preoperatively or intraoperatively. Consistent with previous studies, baseline visual acuity remains the strongest predictor of final visual outcomes in endophthalmitis [15, 16, 20]. A positive vitreous culture reflects higher microbial load or virulence, driving greater intraocular inflammation and structural damage, and thus worse outcomes [21]. RD, whether pre-existing or discovered intraoperatively, remains a particularly severe clinical condition. Wang et al. [22] reported poor outcomes in such cases, with only 37.5% achieving a final visual acuity of 20/400 or better, and 31.3% ultimately requiring enucleation or evisceration.

In eyes with active endophthalmitis, induction of posterior vitreous detachment and peripheral vitreous shaving is theoretically associated with an increased risk of postoperative RD. Our overall RD rate was 8.9%, similar to that reported in the EVS (7.8%) [23]. By comparison, previous studies reported higher rates following 23-gauge core vitrectomy alone (21.6%) [24], whereas 23-gauge complete vitrectomy for maximal microbial clearance had lower rates (12%) [25]. These observations suggest that factors beyond the extent of vitrectomy may contribute to RD risk. Conversely, post-cataract endophthalmitis was associated with a more favorable prognosis than other etiologies, such as trauma or bleb-associated infections, likely due to less virulent organisms and earlier detection [26]. Additionally, pseudophakic eyes provide improved intraoperative visualization, facilitating a more complete vitrectomy and precise delivery of antibiotics.

In our cohort, eyes that underwent 27-gauge vitrectomy achieved numerically better final BCVA compared with 23- and 25-gauge groups. This may reflect baseline differences, including a higher proportion of early intervention (≤ 3 days; 85.7% vs. 48.4%), lower RD incidence (7.1% vs. 22.6%), and fewer positive microbial cultures (20% vs. 51.7%), rather than intrinsic superiority. Previous studies have shown that the 27-gauge system is as safe and effective as the 25-gauge system for ERM [27], primary rhegmatogenous RD [28] and diabetic tractional RD [29].

Theoretically, the advantages of 27-gauge vitrectomy, including smaller incisions [7], reduced surgical trauma [27], faster wound healing [9], and a lower risk of postoperative hypotony [9, 30], are especially beneficial in infected, inflamed eyes with compromised tissue integrity. Additionally, enhanced fluidics and higher cutting rates enable efficient [6] and controlled removal of purulent vitreous material, minimizing collateral damage to intraocular structures [31]. Consistent with prior studies [32–34], these findings support 27-gauge vitrectomy as a feasible option in endophthalmitis. Taken together, our data, despite potential selection and severity biases, suggest that 27-gauge vitrectomy appears not to be inferior to 23- or 25-gauge systems in endophthalmitis management. However, no definitive claim of superiority can be made. Further prospective validation is warranted.

Although prior studies linked early vitrectomy to better outcomes through timely infection control and reduced intraocular toxicity [35, 36], our study did not find a statistically significant association between surgical timing and final visual outcomes (median logMAR BCVA: 1.78 [IQR, 0.30–2.30] vs. 2.34 [IQR, 0.63–2.90], for ≤ 3 vs. > 3 days; *P* = 0.230). This discrepancy may be explained by several factors, including the heterogeneity of disease etiologies, and variation in the definition of “early” vitrectomy across studies; for example, the EVS defined early intervention as within six hours, while more recent studies often use broader criteria (e.g., within 24–48 h) [37]. In addition, advances in intravitreal antibiotic therapy and vitrectomy techniques may have reduced the benefit gap between early and delayed surgery. Routine administration of broad-spectrum intravitreal antibiotics at diagnosis may have helped control infection promptly, irrespective of surgical timing. In summary, while early vitrectomy is biologically plausible and clinically reasonable, its definitive impact on visual outcomes in the modern small-gauge era remains to be fully established, highlighting a knowledge gap that necessitates prospective investigation.

Postoperative complications were not uncommon, reflecting the challenges of managing exogenous endophthalmitis. The most frequent complication was ERM formation (20%), linked to intraocular inflammation and surgical trauma that stimulate retinal gliosis [38]. CME and ocular hypertension each occurred in 13.3%. CME likely results from persistent inflammation and disruption of blood-retinal barrier, both common sequelae in infectious uveitis and following vitrectomy [39]. Ocular hypertension may arise secondary to silicone oil use, steroid response, or trabecular meshwork dysfunction [40]. Hypotony and secondary glaucoma were less common (4.4% each), but both remain vision-threatening if not properly managed. Two eyes developed phthisis bulbi, neither receiving silicone oil tamponade, suggesting a potential protective role of tamponade in severely compromised eyes [41, 42]. One patient required evisceration due to uncontrolled MRSA infection, highlighting the aggressive nature of certain pathogens and the importance of early pathogen identification and targeted therapy. Collectively, these findings emphasize the necessity for vigilant postoperative surveillance.

Strength of our study lies in its real-world, multi-etiology cohort extending beyond the EVS’s post-cataract scope, enabling evaluation of modern approaches, including 27-gauge microincision vitrectomy. Nonetheless, our study has several limitations. As a single-center retrospective study, this analysis is inherently subject to potential selection bias and limited generalizability. Variability in surgical timing and techniques, influenced by individual surgeon discretion, may also introduce confounding factors. Future multicenter prospective studies are warranted to validate our findings and further refine clinical guidelines regarding the optimal timing and technique of PPV in the management of exogenous endophthalmitis. Furthermore, the exploratory analyses for visual outcomes lacked multiplicity adjustment; thus, these hypothesis-generating findings should be interpreted with caution and warrant further validation.

In conclusion, modern small-gauge PPV remains an effective intervention for exogenous endophthalmitis, offering meaningful visual recovery in a substantial proportion of patients. Early recognition, appropriate patient selection, and individualized surgical strategies are crucial for optimizing clinical outcomes.

## Supplementary Information

Below is the link to the electronic supplementary material.

Supplementary Material 1

Supplementary Material 2

## Data Availability

No datasets were generated or analysed during the current study.
