# Supplementary material for: Long-term outcomes and factors associated with visual recovery after small-gauge vitrectomy for infectious endophthalmitis: a 10-year experience at a single tertiary center in Taiwan
Source: Graefes Arch Clin Exp Ophthalmol. 2026 May 14;264(10):3259–68. doi: 10.1007/s00417-026-07279-0 (PMC13635020; doi:10.1007/s00417-026-07279-0)
Supplement: Supplementary file 1 [file 417_2026_7279_MOESM1_ESM.docx]

**Supplement Table 1.** Etiology of infection and pathogens

| Cause of endophthalmitis, no. (%) |  |
| --- | --- |
| Cataract surgery | 34 (75.6%) |
| PPV | 6 (13.3%) |
| Intravitreal injection | 2 (4.4%) |
| Trauma | 1 (2.2%) |
| Bleb needling | 1 (2.2%) |
| Trabeculectomy | 1 (2.2%) |
| Vitreous culture performed, no. (%) | 39 (86.7%) |
| Culture result, no. (%) (n=39) |  |
| Growth | 17 (43.6%) |
| No growth | 22 (56.4%) |
| Culture pathogen, no. (%) (n=17) |  |
| Staphylococcus epidermidis | 7 (41.2%) |
| Streptococcus pneumoniae | 2 (11.8%) |
| Coagulase-negative Staphylococci | 2 (11.8%) |
| Enterococcus faecalis | 2 (11.8%) |
| Viridans streptococci | 1 (5.9%) |
| Unidentified Gram positive bacillus | 1 (5.9%) |
| MRSA | 1 (5.9%) |
| Pseudomonas aeruginosa | 1 (5.9%) |

MRSA, methicillin-resistant Staphylococcus aureus

**Supplement Table 2.** Surgical outcomes and complication

| Final visual acuity (logMAR), median (IQR) | 1.78 (0.35 - 2.90) |
| --- | --- |
| Lensectomy or IOL removal when PPV, no. (%) |  |
| Lensectomy | 3 (6.7%) |
| IOL removal | 9 (20%) |
| Vitreous tamponade after PPV, no. (%) |  |
| Balanced Salt Solution | 29 (64.4%) |
| Silicone oil | 15 (33.3%) |
| Sulfur hexafluoride | 1 (2.2%) |
| Retinal detachment (intraoperative find), no. (%) | 7 (15.6%) |
| Retinal break, no. (%) | 7 (15.6%) |
| Complications |  |
| Epiretinal membrane, no. (%) | 9 (20%) |
| Post-operative ocular hypertension, no. (%) | 6 (13.3%) |
| Cystoid macular edema, no. (%) | 6 (13.3%) |
| Post-operative retinal detachment, no. (%) | 4 (8.9%) |
| Hypotony, no. (%) | 2 (4.4%) |
| Secondary glaucoma , no. (%) | 2 (4.4%) |
| Phthisis bulbi, no. (%) | 2 (4.4%) |
| Neovascular glaucoma, no. (%) | 1 (2.2%) |
| Cornea decompensation, no. (%) | 1 (2.2%) |
| Second surgery, no. (%) | 12 (26.7%) |
| Remove silicone oil | 2 (4.4%) |
| PPV | 3 (6.7) |
| PPV+SB | 2 (4.4%) |
| TSCPC | 1 (2.2%) |
| Anterior chamber irrigation | 1 (2.2%) |
| Cataract surgery or Secondary IOL insertion | 2 (4.4%) |
| Evisceration | 1 (2.2%) |
| Third surgery, no. (%) |  |
| PPV | 2 (4.4%) |

IOL, intraocular lens; PPV, pars plana vitrectomy; SB, sclera buckling; TSCPC, Transscleral cyclophotocoagulation;
